# Unsupervised construction of computational graphs for gene expression data with explicit structural inductive biases

**DOI:** 10.1093/bioinformatics/btab830

**Published:** 2021-12-09

**Authors:** Paul Scherer, Maja Trębacz, Nikola Simidjievski, Ramon Viñas, Zohreh Shams, Helena Andres Terre, Mateja Jamnik, Pietro Liò

**Affiliations:** Department of Computer Science and Technology, University of Cambridge, Cambridge, CB3 0FD, UK; Department of Computer Science and Technology, University of Cambridge, Cambridge, CB3 0FD, UK; Department of Computer Science and Technology, University of Cambridge, Cambridge, CB3 0FD, UK; Department of Computer Science and Technology, University of Cambridge, Cambridge, CB3 0FD, UK; Department of Computer Science and Technology, University of Cambridge, Cambridge, CB3 0FD, UK; Department of Computer Science and Technology, University of Cambridge, Cambridge, CB3 0FD, UK; Department of Computer Science and Technology, University of Cambridge, Cambridge, CB3 0FD, UK; Department of Computer Science and Technology, University of Cambridge, Cambridge, CB3 0FD, UK

## Abstract

**Motivation:**

Gene expression data are commonly used at the intersection of cancer research and machine learning for better understanding of the molecular status of tumour tissue. Deep learning predictive models have been employed for gene expression data due to their ability to scale and remove the need for manual feature engineering. However, gene expression data are often very high dimensional, noisy and presented with a low number of samples. This poses significant problems for learning algorithms: models often overfit, learn noise and struggle to capture biologically relevant information. In this article, we utilize external biological knowledge embedded within structures of gene interaction graphs such as protein–protein interaction (PPI) networks to guide the construction of predictive models.

**Results:**

We present Gene Interaction Network Constrained Construction (GINCCo), an unsupervised method for automated construction of computational graph models for gene expression data that are structurally constrained by prior knowledge of gene interaction networks. We employ this methodology in a case study on incorporating a PPI network in cancer phenotype prediction tasks. Our computational graphs are structurally constructed using topological clustering algorithms on the PPI networks which incorporate inductive biases stemming from network biology research on protein complex discovery. Each of the entities in the GINCCo computational graph represents biological entities such as genes, candidate protein complexes and phenotypes instead of arbitrary hidden nodes of a neural network. This provides a biologically relevant mechanism for model regularization yielding strong predictive performance while drastically reducing the number of model parameters and enabling guided *post-hoc* enrichment analyses of influential gene sets with respect to target phenotypes. Our experiments analysing a variety of cancer phenotypes show that GINCCo often outperforms support vector machine, Fully Connected Multi-layer Perceptrons (MLP) and Randomly Connected MLPs despite greatly reduced model complexity.

**Availability and implementation:**

https://github.com/paulmorio/gincco contains the source code for our approach. We also release a library with algorithms for protein complex discovery within PPI networks at https://github.com/paulmorio/protclus. This repository contains implementations of the clustering algorithms used in this article.

**Supplementary information:**

Supplementary data are available at *Bioinformatics* online.

## 1 Introduction

Gene expression data are commonly used at the intersection of cancer research and machine learning as it is seen as a crucial component towards understanding the molecular status of tumour tissue. In its most common form, an observation of gene expression data is presented as a *k*-dimensional feature vector of continuous values after normalization of the raw data where each element of the vector corresponds to the expression level of a particular gene in the sample. Classically, this representation is directly used to learn a prediction model for tasks such as cancer disease subtype classification or as part of a larger system integrating data from multiple modalities (Esteva *et al.*, 2019; Simidjievski *et al.*, 2019).

The high dimensionality and noisiness of the gene expression data pose significant problems to learning algorithms. Coupled with the comparatively low number of observations, this high dimensionality causes models to overfit, learn noise and struggle to capture any biologically relevant information (Esteva *et al.*, 2019). As a result, practitioners commonly aim to constrain model complexity by incorporating various approaches for regularization including dimensionality reduction and use of prior biological knowledge to inductively bias models towards learning representations with favourable characteristics (Cawley and Talbot, 2006; Dutil *et al.*, 2018; Gustafsson *et al.*, 2005; Simidjievski *et al.*, 2019). Our research uses prior knowledge to focus on the incorporation of gene interaction networks as external priors into the predictive model in order to guide the learning process. The overall goal of applying network-based analysis to personal genomic profiles is to identify network modules that are both informative of cancer mechanisms and predictive of cancer phenotypes. A survey which describes some of these approaches can be found in Zhang *et al.* (2017). However, many of these methods are handcrafted to address very specific case studies and typically they are not end-to-end differentiable which is the focus of this study.

In this work, we propose a method for automated construction of predictive neural network models that build upon structures discovered within gene interaction networks. More specifically, we utilize topological clustering algorithms chiefly used for the discovery of protein complexes and functional modules within protein–protein interaction (PPI) networks to define the structure of factor graphs in an unsupervised manner. This deterministic procedure produces sparse computational graph models which relate genes to named protein complexes, structurally parameterizing individual functions for the ‘activity’ of each complex based on an input gene expression profile. Given such computation graphs, further connecting the complex activities to cancer phenotypes defines a supervised predictive model akin to a sparsely connected artificial neural network, which maps the activity patterns of higher level functional modules (protein complexes) to cancer phenotypes via the original gene expression data.

**Fig. 1.**
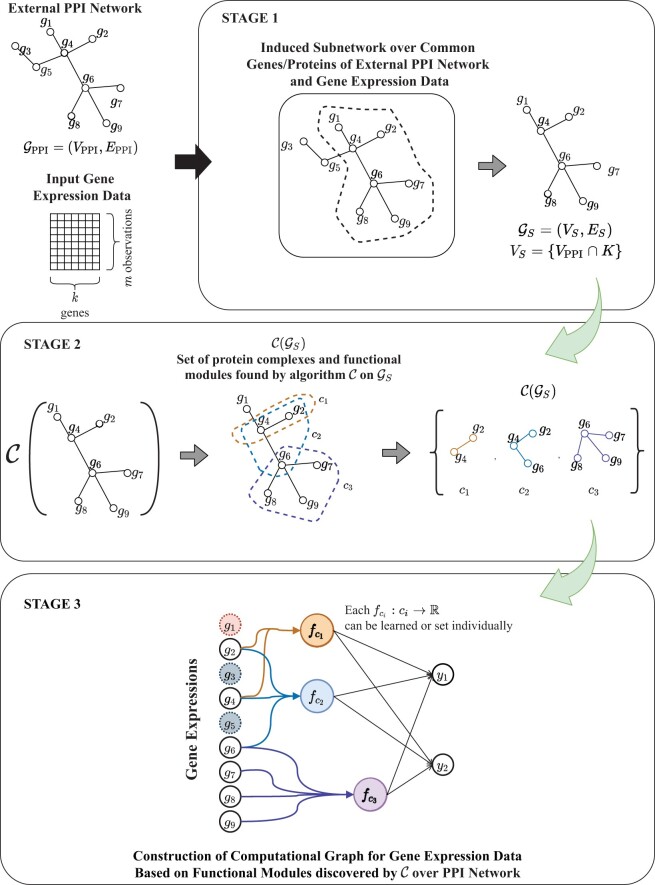
An overview of our procedure for incorporating PPI network based protein complex discovery and constructing computational graphs for gene expression analysis. GINCCo’s procedure for model construction is best described in three stages: (i) induction of the case study specific sub-graph $G_{S}$ common to the input gene expression dataset (for set of *k* genes *K*) and the external PPI network which will be used for the (ii) unsupervised discovery of the protein complexes that act as biologically relevant higher level modules of the inputs and (iii) the use of the clusterings $C(G_{S})$ to construct a bipartite factor graph between the gene expressions and the protein complexes and extending the use of the graph in the predictive model that transitively maps the gene expressions to phenotypes via the protein complex activities. In the final computational graph model, we can see blue genes which are excluded as a result of extracting the case specific study graph, and red genes which are excluded as a result of clustering process on $G_{S}$

Our approach effectively constrains the hypothesis space via explicit structural biases obtained through unsupervised analyses of network biology entities. As a result, this provides a biologically relevant mechanism for model regularization, resulting in structurally constrained models that yield competitive predictive performance with significantly lower number of model parameters and offer insights into the expression patterns of phenotype relevant complexes. Figure 1 features a simplified diagram of this process over an input genomic profile dataset and a toy interaction network used to construct the topology of the computational graph.

## 2 Materials and methods

The proposed method, which we will refer to as Gene Interaction Network Constrained Construction (*GINCCo*), incorporates prior biological knowledge embedded within the structure of external PPI networks and protein complexes discovered in these via topological clustering algorithms to construct a bipartite graph between gene expressions and functional modules. This bipartite factor graph serves as the structural foundation for computational graph models that will be further augmented into predictive models for cancer phenotypes. Crucially, this means that the structure of the computational graphs created by GINCCo is defined in a purely unsupervised and deterministic manner over external structured knowledge.

GINCCo’s procedure for constructing the computational graphs is best described in three stages which also correspond to those shown in Figure 1:

- Obtaining a case study specific sub-graph of an external PPI network with the input gene expression data.
- Discovering protein complexes that serve as higher level functional modules within the study specific sub-graph from Step 1.
- Constructing the factor and computational graphs for downstream modelling.

### 2.1 Processing and generating case study PPI networks

Let us assume an input gene expression dataset $X\in {\mathbb{R}}^{m\times k}$ describing *m* patient observations with *k*-dimensional vectors of gene expression values, and *K* represents the set of genes in this expression dataset. Furthermore, let us assume an external PPI network $G_{\text{PPI}}=(V_{\text{PPI}},E_{\text{PPI}})$, such as one from the STRING-DB 9606 *Homo Sapiens* PPI network (Szklarczyk *et al.*, 2019). For our purpose, this PPI network is an unweighted graph with nodes $V_{\text{PPI}}$ labelled by the names of proteins, and no additional node or edge features. We induce a sub-graph of the input network $G_{S}\subseteq G_{\text{PPI}}$. The nodes of $G_{S}$ are the intersection of the common genes in the input gene expression dataset *K* and their products in the PPI network; in other words $V_{S}=K\cap V_{\text{PPI}}$. The induced sub-graph $G_{S}=(V_{S},E_{S})$ is the graph whose vertex set is $V_{S}$ and whose edge set consists of all of the edges in $E_{\text{PPI}}$ that have both endpoints in $V_{S}$. This action is illustrated in the top row of actions in Figure 1. We denote $G_{S}$ our study PPI network since it is the ‘cut out’ of the external PPI network relevant to our case study.

### 2.2 Protein complex discovery

Given the induced study network, we use a topological clustering algorithm C (such as DPCLUS; Altaf-Ul-Amin *et al.*, 2006) to discover protein complexes within the study PPI network $G_{S}$. The aim of the clustering algorithms is to discover protein complexes represented as a set of induced sub-graphs $C(G_{S})=\{c_{1},c_{2},\ldots ,c_{l}\}$, where *l* is the number of complexes discovered by C. The number of protein complexes found, *l*, is not dependent on the user, but rather on the application of the clustering algorithm C upon the input study network. Any appropriate clustering algorithm can be used.

It is worth noting that we specifically chose clustering algorithms that do not partition the graph. In other words, a single protein may be part of multiple complexes. This is to reflect the fact that proteins may be involved in several biological processes and complexes. Moreover, not all proteins in $G_{S}$ will necessarily be assigned to clusters by *C*. We are not arbitrarily forcing all genes to be part of our constructed models, and this acts as a form of feature selection upon the input **X** by $C(G_{S})$.

### 2.3 Computational graph construction and predictive models

The output of the clustering algorithm $C(G_{S})=\{c_{1},c_{2},\ldots ,c_{l}\}$ enables the construction of a bipartite factor graph. Herein, each of the protein complexes is assigned a uniquely labelled node *c_i_* and each protein within the set of proteins involved in one or more complexes is also given a labelled node by their name. Directed edges link proteins to complexes *c_i_* they are a member of. This construction gives the factorization of a parametric function $f_{c_{i}}:c_{i}\to \mathbb{R}$ computed from the proteins involved in *c_i_*. The function $f_{c_{i}}(\cdot )$ can be set by the practitioner or learned as in a neural network.

**Fig. 2.**
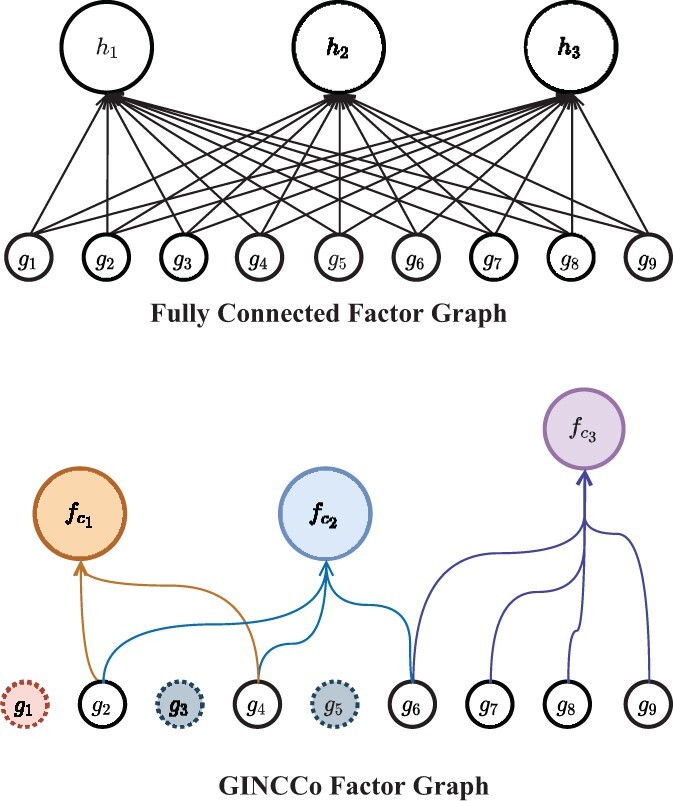
A visual comparison between the factor graphs produced using a FC computational graph as in a standard neural network and that produced by GINCCo using the toy example introduced in Figure  1

The parameterizations $f_{c_{i}}:c_{i}\to \mathbb{R}$ in our proposal are a stark contrast to arbitrarily chosen hidden-state activations $h_{i}:{\mathbb{R}}^{k}\to \mathbb{R}$ found in conventional application of fully connected multi-layer perceptrons (FC MLPs). First, each of the *c_i_* denotes a ‘protein complex activity’, a biologically relevant structure modelled through incorporation of external PPI and topological clustering algorithm, instead of an arbitrarily chosen hidden state node. The proteins that are members of *c_i_*, and only those proteins, affect its activity level $f_{c_{i}}:c_{i}\to \mathbb{R}$, instead of all input features. This is a strong and explicit inductive bias if $f_{c_{i}}$ is learned through a neural network. A visual comparison between the factor graphs of a FC model and that of GINCCo can be seen in Figure 2.

We construct computational graph models for cancer phenotype prediction by further augmenting the current gene/protein to protein complex factor graph to include complete connections between the protein complexes *c_i_* to target nodes gained when encoding the target observations **Y**. As such, each function $f_{c_{i}}:c_{i}\to \mathbb{R}$ computing the individual protein complex ‘activity’ is learned over minimizing the global cross-entropy loss between predicted and the target phenotypes.

### 2.4 Experimental setup

We hypothesized that the knowledge-driven construction of the computational graphs through incorporation of gene interaction networks as prior biological knowledge will yield sparser models and better predictive performance than FC baselines. We tested this hypothesis in parts: comparing model sparsity in terms of number of parameters, comparing predictive performance across datasets and subsequently checking whether GINCCo captures useful signals that cannot be found through random computational graph construction.

In order to evaluate the proposed method for model construction, we used publicly available gene expression data from the METABRIC Breast Cancer Consortium (METABRIC; Curtis *et al.*, 2012) to predict cancer phenotypes with gene expression data. The dataset consists of the mRNA expression data and clinical data of breast cancer patient samples in the METABRIC cohort (Curtis *et al.*, 2012). Herein, we tackle several classification tasks over the 1980 breast cancer patients, representing a particularly large dataset for cancer data research. Each observation is represented by a 24 368-dimensional vector corresponding to the continuous expression values of measured genes. The microarray data were normalized as described in Curtis *et al.* (2012). We evaluate the predictive performance over the proposed methods ability to predict:

- Distance relapse, a binary classification task.
- IntegrativeCluster subtypes (IC10), a 11 class prediction task where observations belong to integrative clusters typified by copy number aberrations (Curtis *et al.*, 2012).
- PAM50 breast tumour cancer subtype (Prat *et al.*, 2010; PAM50), a five class prediction task (Basal, Her2, Luminal A, Luminal Band Normal).

To show that GINCCo can operate across datasets, we also evaluate it on The Cancer Genome Atlas Head–Neck Squamous Cell Carcinoma (TCGA-HNSC) dataset (Cancer Genome Atlas Network, 2015; Rendleman *et al.*, 2019). The HT-Seq count expression data were normalized using the Fragments Per Kilobase of transcript per Million mapped reads method as made available through the National Cancer Institute Genomic Data Commons Data Portal, https://portal.gdc.cancer.gov/ that have been as in Rendleman *et al.* (2019). The dataset contains 528 TCGA-HNSC cases wherein we focus on the 20 501 mRNA expression features. The clinical targets include:

- tumour grade, wherein observations are classified into Grades I–IV based on standards set by the World Health Organization.
- 2-year relapse-free survival, a binary prediction task.

For all prediction tasks, tables of the exact class label distributions are presented in the supplementary materials (Supplementary Appendix SA).

Amongst the considered methods are: majority class classifier (MajorityClass), a support vector machine (SVM) with RBF kernel, a FC two-layer neural network with 1600 hidden layer nodes (this number of hidden nodes was chosen to closely match the number of protein complexes used in GINCCo + DPCLUS, the best performing of the proposed methods), a network regularized FC network (Li and Li, 2008; GraphReg), and our proposed model constructor coupled with a variety of topological clustering algorithms. Each of our models is referred to as GINCCo + C, where C refers to one of: Molecular Complex Detection (MCODE) (Bader and Hogue, 2003), COre-AttaCHment-based method (COACH) (Wu *et al.*, 2009), IPCA (Li *et al.*, 2008) or DPCLUS (Altaf-Ul-Amin *et al.*, 2006) clustering algorithms. These clustering algorithms were chosen on the basis that they are well established, allow overlapping clusters and have deterministic implementations for reproducibility. We also release an open-source library of these implementations alongside this article as described in the availability statement.

MCODE is an agglomerative clustering algorithm for identification of protein complexes given PPI graphs. COACH is an algorithm for identification of protein complexes based on core-attachment structure. DPCLUS is an iterative algorithm for protein-complex identification from interaction graphs. Similarly to MCODE, given a PPI graph, DPCLUS initializes the clusters with the node with the highest weight, identified by analysing node neighbourhoods. Once a cluster is initialized, the algorithm extends it by adding neighbouring nodes that meet predefined criteria of density and cluster-connectivity property. IPCA is a modification of DPCLUS. Similar to DPCLUS, IPCA grows the clusters based on the topological structure of the underlying interaction graph by searching for small-diameter sub-graphs that meet certain cluster connectivity-density property. In contrast to DPClus that re-computes the node weights each time a sub-graph is removed, IPCA computes these weights once at the beginning and uses them for the whole process. The hyperparameters of the clustering algorithms were set to their default values.

The SVM’s hyperparameters were kept the same at *C *=* *1.0 and a scaled *γ* value. The FC MLP and the computational graphs of GINCCo were trained through optimization of the cross-entropy loss. The loss was optimized using Adam (Kingma and Ba, 2014) with a mini batch size of 32 and 500 epochs and a learning rate of 0.0001. The weight parameters were initialized using the Xavier uniform initialization (Glorot and Bengio, 2010).

For each task, we compare the methods over the average performance of five repeated class-stratified train and hold-out test splits with 80:20 train:test set ratios. We use a quarter of each training-set split to produce a validation set for early stopping. The performance of each model was compared with respect to average balanced classification accuracy (B-ACC) and weighted area under receiver operator characteristic (W-AUC) over each of the five splits in the tasks to account for any class imbalances. To compute the W-AUC, we averaged the one-versus-rest scores for each label weighted by the class label distribution. For completeness, we have included tables for the comparative analysis of unbalanced accuracy, weighted precision, weighted recall and weighted *F*-scores which can be found in the Supplementary Appendix SC.

## 3 Results

### 3.1 Factor graphs produced by GINCCo are considerably sparser than FC network models

The computational graph models produced by GINCCo innately incorporate biological knowledge of the PPI network and the multi-protein modules discovered through $C(G_{S})$ over the study network. The resulting bipartite factor graphs between the gene expressions and protein complex activities $f_{c_{i}}:c_{i}\to \mathbb{R}$ are considerably sparser than their FC counterparts as $\forall c_{i}\in C(G_{S}),|c_{i}|\leq k$ by design and often $|c_{i}|\ll k$ as seen in Table 1. Table 1 describes the number of edges (parameters) in the bipartite graph produced by GINCCo and a given clustering algorithm C(·) on the study network created with STRING and the 24 368 genes in the METABRIC dataset. Table 2 describes descriptive statistics of the clusters obtained by each clustering algorithm on the study network. This is compared against the number of edges formed in the FC counterpart with the equal number of hidden activities *h_i_*; a visual comparison can be found in Figure 2. Table 1 shows how GINCCo models have orders of magnitude less parameters than their FC counterparts, and we will show that despite this the models still perform competitively in predictive tasks and bring additional benefits.

**Table 1.** Number of parameters used in equally dimensioned FC MLP network and the proposed method using different clustering methods to automatically discover protein complexes and their members on the STRING 9606 PPI network and the 24 368 genes measured in METABRIC

Method	MCODE (40 clusters)	COACH (4108 clusters)	IPCA (5744 Clusters)	DPCLUS (1562 clusters)
FC MLP	974 720	100 103 744	139 969 792	38 062 816
GINCCo	14 537	1 431 338	2 800 267	19 545

### 3.2 Empirical results show integration of prior biological knowledge yields strong predictive performance

The main comparative results are summarized in Table 2 for the METABRIC and TCGA-HNCS datasets. The results show that all variations of the computational graph models produced by GINCCo perform strongly against both the SVM and FC MLP baselines.

**Table 2.** Average percentage balanced accuracy (B-ACC) and W-AUC with SDs over five repeated train and holdout test evaluations using all of the gene expression features of METABRIC and TCGA-HNCS

Method	METABRIC						TCGA-HNCS			
	Distance relapse		PAM50		IC10		Tumour grade		2-year relapse-free survival	
	B-ACC	W-AUC	B-ACC	W-AUC	B-ACC	W-AUC	B-ACC	W-AUC	B-ACC	W-AUC
MajorityClass	50.00 ± 0.00	0.50 ± 0.00	20.00 ± 0.00	0.50 ± 0.00	9.09 ± 0.00	0.50 ± 0.00	25.00 ± 0.00	0.50 ± 0.00	50.00 ± 0.00	0.50 ± 0.00
SVM	54.43 ± 1.85	0.54 ± 0.02	72.21 ± 3.07	0.94 ± 0.01	55.72 ± 3.79	0.95 ± 0.01	39.35 ± 4.28	0.67 ± 0.04	56.59 ± 4.83	0.57 ± 0.05
FC MLP	56.92 ± 2.65	0.57 ± 0.03	74.65 ± 3.60	0.94 ± 0.01	66.32 ± 1.99	0.95 ± 0.01	34.29 ± 3.53	0.66 ± 0.04	58.14 ± 4.23	0.58 ± 0.05
GraphReg	49.86 ± 1.05	0.50 ± 0.01	22.57 ± 2.71	0.82 ± 0.01	9.09 ± 0.00	0.83 ± 0.01	27.63 ± 3.25	0.64 ± 0.02	55.42 ± 2.35	0.55 ± 0.02
GINCCo + MCODE	56.65 ± 1.86	0.57 ± 0.02	73.52 ± 2.71	0.93 ± 0.01	57.77 ± 1.73	0.93 ± 0.01	36.93 ± 10.14	0.64 ± 0.03	55.43 ± 2.87	0.55 ± 0.03
GINCCo + COACH	56.73 ± 0.98	0.57 ± 0.01	74.97 ± 3.27	0.95 ± 0.01	63.04 ± 2.98	0.95 ± 0.01	39.38 ± 11.48	0.65 ± 0.03	56.79 ± 3.49	0.57 ± 0.03
GINCCo + IPCA	57.13 ± 1.47	0.57 ± 0.01	74.62 ± 4.55	0.94 ± 0.01	62.26 ± 4.51	0.94 ± 0.01	37.36 ± 9.54	0.63 ± 0.03	55.56 ± 3.39	0.55 ± 0.03
GINCCo + DPCLUS	57.27 ± 1.80	0.57 ± 0.02	75.97 ± 4.59	0.97 ± 0.01	70.43 ± 3.68	0.97 ± 0.00	39.09 ± 9.96	0.67 ± 0.03	57.17 ± 4.42	0.57 ± 0.04

More specifically, GINCCo + DPCLUS performs competitively overall making an especially substantial gain in IC10 subtype prediction. Performing a pairwise frequentist correlated *t*-test (Benavoli *et al.*, 2017) shows that GINCCo + DPCLUS has statistically significant performance gains across all tasks compared with MajorityClass and GraphReg methods but is not significant against the other methods except on IC10 subtype prediction (see Supplementary Appendix SD). However, this result is still good as it comes in spite of the fact that the GINCCo + DPCLUS model contains <0.05% of the number of parameters used in the FC MLP (see Table 1). Furthermore, GINCCo models provide additional features pertaining biologically relevant insights that are not possible with the other methods as we show in Section 3.3.

We attribute the strong performance of GINCCo to two related advantages over FC networks. First, GINCCo’s sparser model complexity allows more ‘weight’ to be assigned to each of the input signals used. Similarly, the sparse connectivity also helps generalizability in a similar way to the dropout regularization method. However, in contrast, the connectivity of GINCCo graph is set, explicit and realized through incorporation of prior knowledge rather than being random and ephemeral. This brings us to the second advantage of GINCCo—the structure of the computational graphs, and thus the representations, explicitly incorporate biological knowledge of protein complex membership as intermediate states. In other words, they are not ‘hidden’ nodes with arbitrary meaning. The learned activities of the protein complexes are explicitly factorized to the gene expression measurements of the genes/proteins that have a membership in the complex. To show that GINCCo benefits from both of the previously mentioned advantages and not only from the first advantage of regularization via sparse connections, we demonstrate that the performance of GINCCo + DPCLUS outperform computational graphs constructed through random processes (RC MLP-R and RC MLP-M).

The differing performances on the choice of clustering algorithm C(·) reflect the different assumptions made by researchers on what topological structures within $G_{S}$ contain protein complexes. MCODE and DPCLUS exhibit stricter rules on complex candidates with fewer, smaller and more tightly knit clusters than either COACH or IPCA as in Table 3. This may be interpreted as these two methods constraining the hypothesis space more and incorporating ‘more’ expert knowledge which is helpful to the classification tasks. Naturally, GINCCo is agnostic to the choice of C(·), therefore various combinations or set complexes may be explored in further work.

**Table 3.** Descriptive statistics of the protein complexes discovered via the topological clustering of the study PPI network $G_{S}$ induced from the STRING PPI network and METABRIC

Statistic	MCODE	COACH	IPCA	DPCLUS
Number of protein complex	40	4108	5744	1562
Maximum cluster size	1555	2684	639	359
Minimum cluster size	3	4	5	2
Average cluster size	363.43	348.43	487.51	12.51

### 3.3 Experiments against randomly structured computational graphs show GINCCo models capture useful parameterizations

As the structure of the computational graphs is driven largely by the structure of the external PPI network and the number/members of the protein complexes discovered, we check that GINCCo graphs actually capture biologically relevant information. Naturally, the structure of the PPI network itself is explained and justified by the maintainers/proposers/curators of the databases. Similarly, the biological relevance of the clustering algorithms used on the PPI networks is also reasoned and justified within each of the original papers. Hence, our task here is to find whether the computational graphs constructed through GINCCo obtain better scores than the SVMs and FC-MLP because the structure and learned activity functions capture meaningful biological relationships.

We test this with two approaches to generate randomly connected computational graph models, referred to as RC MLP-R and RC MLP-M. For RC MLP-R, we construct computational graphs with a random number of ‘discovered protein complexes’ and a random number of connections attributing protein memberships to clusters. The random numbers are drawn from a uniform distribution between l∈[30,6000] for the number of protein complexes (this range was chosen to roughly reflect the number of protein complexes found in the chosen clustering algorithms on the STRING-DB PPI network; see Table 3) and u∈[1,l*k] random protein to complex connections. For RC MLP-M models, we preserve the number of complexes and connections used in GINCCo + DPCLUS but perturb the connections. Hence, $l_{\text{RCMLPM}}=l_{\text{DPCLUS}}$ and $u_{\text{RCMLPM}}=u_{\text{DPCLUS}}$, translating to *l *=* *1562 and *u *=* *19 545 for METABRIC tasks. For an empirical evaluation, 100 instances of such random computational graphs were constructed to obtain a Monte-Carlo aggregate mean score across the same repeated train-test evaluation described in Section 2.4. Results are shown in Table 4.

**Table 4.** B-ACC and W-AUC with SDs over five repeated train/test evaluations using all of the gene expression features of METABRIC and TCGA-HNCS

Method	METABRIC						TCGA-HNCS			
	Distance relapse		PAM50		IC10		Tumour grade		2-year relapse-free survival	
	B-ACC	W-AUC	B-ACC	W-AUC	B-ACC	W-AUC	B-ACC	W-AUC	B-ACC	W-AUC
FC MLP	56.92 ± 2.65	0.57 ± 0.03	74.65 ± 3.60	0.94 ± 0.01	66.32 ± 1.99	0.95 ± 0.01	34.29 ± 3.53	0.66 ± 0.04	58.14 ± 4.23	0.58 ± 0.05
RC MLP-R	56.91 ± 0.78	0.57 ± 0.01	72.06 ± 6.55	0.93 ± 0.04	57.25 ± 10.03	0.92 ± 0.06	38.02 ± 3.26	0.64 ± 0.05	54.86 ± 1.58	0.54 ± 0.02
RC MLP-M	55.25 ± 1.56	0.55 ± 0.02	64.87 ± 8.79	0.92 ± 0.05	54.10 ± 6.68	0.91 ± 0.04	35.45 ± 2.45	0.66 ± 0.01	54.15 ± 1.87	0.54 ± 0.02
GINCCo + DPCLUS	57.27 ± 1.80	0.57 ± 0.02	75.97 ± 4.59	0.97 ± 0.01	70.43 ± 3.68	0.97 ± 0.00	39.09 ± 9.96	0.67 ± 0.03	57.17 ± 4.42	0.57 ± 0.04

From RC MLP-R results, we can see how on average a sparse randomly structured instantiation of a computational graph model does not outperform the FC model or GINCCo + DPCLUS, often performing significantly worse on multi-label tasks and with highly variable outputs. This suggests that the unguided random sparsification does not lead to better results. This is further compounded by the results from RC MLP-M which show that despite the preservation of the number of ‘complexes’ and connections of GINCCO + DPCLUS, the randomizations of the connections hurt the performance. Moreover, this suggests that the inductive biases offered by explicit factorizations of genes and protein complexes via validated biologically inspired clustering algorithms drastically reduce the number of model parameters, perform competitively and also enable guided *post-hoc* enrichment studies of target relevant functional modules, as we show next.

A benefit of the deterministic and explicit factorization of the parametric activity functions of named protein complexes (and potentially other higher level modules such as pathways) presents interesting opportunities for introspective analyses of the models. Each of the candidate protein complexes may be functionally analysed through gene set enrichment analyses that can provide insights into the patterns of ‘active’ functional modules with respect to the input gene expressions and the disease phenotypes. A preliminary *post-hoc* analysis to identify functionally relevant complex candidates with trained GINCCo models is presented in the supplementary materials (Supplementary Appendix SB). In particular, we leveraged Integrated Gradients (Sundararajan et al., 2017), a gradient-based attribution method, to estimate the importance of intermediate protein complex nodes in the computation of the target values. We then ranked the protein complexes according to their importance to the prediction task and performed functional enrichment analysis using Enrichr (DisGeNET) to identify enriched pathways. For classification of PAM50 on the METABRIC dataset with GINCCo + DPCLUS, we found that the top enriched pathways for the most important complex candidates are (i) malignant neoplasm of the breast (*q*-value: 2.4e-21) and (ii) breast carcinoma (*q*-value: 8.35e-21). These results suggest that the protein complexes identified by DPCLUS are biologically meaningful and further support our choice for incorporating them as structural inductive biases in our model. More generally, this result shows the potential of GINCCo to help identify functionally relevant gene-sets given specific phenotype targets and to enable their study through functional enrichment analyses.

## 4 Related work and discussion

This work is focussed on the utilization of prior biological knowledge embedded within the topologies of interaction networks to guide the construction of predictive models. Therefore, it is related to several other approaches that incorporate inductive biases from the topologies of external molecular networks into neural networks (and other modelling approaches) as well as end-to-end differentiable models. More closely, GINCCo relates to Knowledge-Primed Neural Networks (KPNNs; Fortelny and Bock, 2020), that explicitly incorporate biological networks in the design of the neural network architecture. Similarly to GINCCo, the input nodes correspond to genes (or proteins), but the hidden units of the neural network correspond to various signalling proteins and transcription factors. This, in turn, leads to an accurate and interpretable predictive model for single-cell analysis. However, in order to produce such models, KPNNs require topological data in the form of directed acyclic graphs with explicitly defined regulatory mechanisms. In contrast, GINCCo is more general in this respect, since it is not constrained by the type nor completeness of the structural prior. This allows for incorporating (and combining) different topological data for various applications including, but not limited to single-cell analysis, such as cancer sub-type identification/classification.

Other similar approaches have been proposed recently exploiting knowledge of biological pathways to create sparse neural network models. PASNet (Hao *et al.*, 2018) and P-NET (Elmarakeby *et al.*, 2020) incorporate pathway information for survival prediction in glioblastoma multiforme and for stratification of prostate cancer patients, respectively. These approaches are all closely related to GINCCo. However, P-NET requires careful handcrafted construction of the architecture as well as manual curation of certain layers. In contrast, GINCCo is more general, fully automated and leads to substantially smaller models. Moreover, the clustering step in GINCCo is independent; therefore, it can handle various types of domain-knowledge (including pathways). Similarly, PASNet refers to a sparse neural network that also relies on knowledge-based structural biases, by incorporating pathway information. In that, it is similar to GINCCo, however, instead of ‘learning’ the second hidden layer from the constructed clusters (as in GINCCo), PASNet explicitly maps the pathways. Therefore, in that respect, GINCCo is more general, since it does not explicitly rely on known pathway sets.

In broader terms, GINCCo follows a long tradition of methods that incorporate biological knowledge through feature selection and extraction. In particular, it relates to embedded techniques (Hira and Gillies, 2015) that simultaneously select subsets of the original gene features and build a predictive model such as SVM-RFE (Guyon *et al.*, 2002) and LASSO (Ma *et al.*, 2007). GINCCo distinguishes itself here in that it performs the selection and model construction in a completely automated, deterministic and unsupervised manner; this can be seen as a pre-processing step allowing GINCCo to scale immensely and study factor graphs without the influence of task-specific optimization dictating the shape of the models.

Incorporating topological inductive biases can also be performed with network regularization methods as seen in Gustafsson *et al.* (2005), Li and Li (2008) and Min *et al.* (2018). Herein, methods such as graph Laplacian regularization work on regularizing the coefficients of linear models such that they are similar for terms that are connected within the incorporated network. We have included the method proposed by Li and Li (2008) within our comparative analysis in the previous section. A benefit of graph regularization as a method for incorporating prior knowledge is that it does not require a separate clustering stage as in GINCCo. However, this comes at the cost of not being able to study the potential gene sets (in our case protein complexes) for functional relevance, such as post-training analysis using functional enrichment analysis in the Supplementary Appendix SB. Furthermore, there is a subtle but important difference in the aims of our method and graph regularization methods in terms of the inductive bias produced. The graph Laplacian regularization is a summation of the smoothness terms on the variables to encourage similar coefficients on the genes that are connected. In contrast, GINNCo models are inductively biased (quite explicitly) to produce representations based on the subnetworks extracted by the clustering algorithms. Naturally, as graph regularization methods are typically implemented as a regularization term, they can be trivially incorporated into the objective function of GINCCo models as well.

More generally, variations operating on the general network propagation model have found increasing use within research involving network biology (Cowen *et al.*, 2017). Parallel research took place within machine learning communities on graph neural networks which impose a graph constrained inductive bias onto the representations learned in neural networks mostly on social networks (Belkin and Niyogi, 2001; Defferrard *et al.*, 2016; Kipf and Welling, 2017). Such neural networks are characterized by *graph convolutional* operators that serve as useful inductive biases for learning representations of nodes and other graph substructures.

The clear biological motivations (Cowen *et al.*, 2017) behind the network propagation model and its parallels to graph neural network(GNN) models quickly inspired a succession of works aimed at using GNNs architectures on gene expression data. Rhee *et al.* (2018) use a ChebNet (Defferrard *et al.*, 2016) variant with a relation network (Santoro *et al.*, 2017) to impose a PPI network upon each of the genomic profiles. Here, each of the gene expression values is mapped onto a copy of the PPI structure. This was used to classify genomic profiles from the TCGA into PAM50 classifications for breast cancer subtype classifications. Chereda *et al.* (2019) provided a simpler architecture solely using a ChebNet on the gene expression values mapped on a PPI network to predict metastasis. The published results on metastasis show that their proposed method is marginally better (1–2%) than their random forest and FC neural network baselines. This naturally raises the question of whether the positive performance published in Rhee *et al.* (2018)’s hybrid model comes primarily from their GNN or relational network component or the combination of both.

A series of closely related research (Bertin *et al.*, 2019; Dutil *et al.*, 2018; Hashir *et al.*, 2019) has studied integrating various *gene interaction networks* such as PPI, gene regulatory, transcription regulation, etc. as masking measures over the features to impose an inductive bias. Experiments were carried out on single-gene inference tasks (Dutil *et al.*, 2018) and a cancer phenotype prediction task (Bertin *et al.*, 2019). The usage of the network information was deemed useful for the single-gene inference task, but also important negative results in some experiments where the prior knowledge of a curated graph was about as useful as a randomly connected graph was also reported—highlighting the importance of choosing the ‘right’ graph as prior knowledge. On the phenotype prediction task, using graphs as a mask over the gene expressions as prior knowledge was unable to beat a baseline multilayer perceptron on the same task (Bertin *et al.*, 2019).

The work on applying GNNs to incorporate prior network information to genomic data tasks is a nascent and valid general approach to the problem. However, the differential graph convolution and pooling operations as used in previous work, are not best suited to learn biologically useful subnetworks for the predictive model within the small datasets that are available now. The classic graph convolutional operations used in Rhee *et al.* (2018) and Chereda *et al.* (2019) consider higher level node aggregations of all its neighbours with equal weight. When the nodes of the GNNs are genes superimposed onto a gene interaction graph (let us say a PPI network) the resulting node feature only consists of the gene expression scalar. The feature propagation mechanism between neighbours creates a bottleneck when every node aggregates messages from its neighbours (Alon and Yahav, 2020). Each of the scalars is simply mixed into another scalar value through the aggregation. Differentiably learned pooling methods require an increasing number of samples to learn ‘useful’ higher level representations, which are not explicitly related to a biologically relevant entities. Furthermore, pooling methods have recently been shown to have inherent limitations in actually capturing local receptive fields better than random cluster assignments (Mesquita *et al.*, 2020).

In contrast, the models created through our proposed framework forego learning ‘hidden’ higher level representations by explicitly factorizing the transitive relationship between gene expressions, protein complex activity, and phenotypes using PPI networks and deterministic protein complex discovery algorithms. This is done specifically to constrain the hypothesis space of potential models and impose structure using domain knowledge on the scarce data in the gene expression datasets. It relates each gene expression to a named higher level entity, the protein complex and has a function specific weighting that is learned (or set based on the practitioner) through the global optimization scheme over this computational graph. As a result, the signal from each gene expression is not equally weighted, but specific to each complex activity function—signals are even dropped explicitly through the $C(G_{S})$ function if they are not within the scope of study for the computational graph. This is unlike the GNN or a network regularized method which would include all of the input and try to learn something from it even if it were noise. Thus, our method is substantially different and additive on both existing approaches.

## 5 Conclusion

We presented GINCCo, a scalable unsupervised approach to incorporating biological knowledge embedded in the structure of gene interaction networks for automated construction of computational graphs for gene expression analysis. GINCCo has several distinguishing properties. First, it provides a biologically relevant mechanism for model regularization, resulting in structurally constrained models that often yield better predictive performance whilst drastically reducing model parameters and enabling *post-hoc* enrichment analyses. Secondly, GINCCo is scalable and applicable to other tasks beyond the case study presented where explicitly modelling the activities of subnetworks within networks describing prior knowledge can be beneficial to a data analysis task. For example, the computational graphs can be seamlessly incorporated into larger integrative frameworks handling multiple modalities such as the integrative variational auto-encoders in Simidjievski *et al.* (2019) to reduce the complexity of its hypothesis space. Finally, there is no arbitrary decision making on the number of hidden nodes or their biological relevance as in standard MLPs. Each node within our computational graphs is either a gene, a phenotype, or a candidate protein complex. The structure describes a knowledge-directed factorization of the parametric function for the activity of a protein complex based on the expression levels of its constituent gene/proteins. This makes introspective study into the individual contributions and functional roles of entities in the model and patterns as a whole more amenable.

## Data availability

The data underlying this article are publicly available for METABRIC at https://www.cbioportal.org/study/summary?id=brca_metabric and for TCGA-HNCS at https://portal.gdc.cancer.gov/. The TCGA-HNCS dataset version used in this article was derived from Rendleman *et al.* (2019) available in the public domain: https://github.com/mrendleman/ MachineLearningTCGAHNSC-BINF/. The STRING 9606 Human PPI network is publicly available at https://string-db.org/cgi/download. Additionally, any data supporting the conclusions of this article will be shared on reasonable request to the corresponding author. Source code to implementations is made available as in the availability statement on the title page.

## Funding

P.S. was funded by the W.D. Armstrong Fund from the School of Technology at the University of Cambridge. N.S., H.A.T, Z.S., M.J. and P.L. were funded by The Mark Foundation Institute for Integrated Cancer Medicine (MFICM). MFICM is hosted at the University of Cambridge, with funding from The Mark Foundation for Cancer Research (NY, USA) and the Cancer Research UK Cambridge Centre [C9685/A25177] (UK). 

*Conflict of Interest*: none declared. 

## Supplementary Material

btab830_Supplementary_Data
