# Supplementary material for: Unsupervised construction of computational graphs for gene expression data with explicit structural inductive biases
Source: Bioinformatics. 2021 Dec 9;38(5):1320–7. doi: 10.1093/bioinformatics/btab830 (PMC8826027; doi:10.1093/bioinformatics/btab830)
Supplement: btab830_Supplementary_Data [file btab830_supplementary_data.pdf]

*Bioinformatics*
doi.10.1093/bioinformatics/xxxxxx
Advance Access Publication Date: Day Month Year
Manuscript Category

OXFORD

Supplementary Material

# Unsupervised construction of computational graphs for gene expression data with explicit structural inductive biases

**Paul Scherer, Maja Trębacz, Nikola Simidjievski, Ramon Viñas, Zohreh Shams, Helena Andres Terre, Mateja Jamnik and Pietro Liò**

Department of Computer Science and Technology, University of Cambridge, Cambridge, CB3 0FD, United Kingdom

**Abstract**

## 1 Appendix A: Sample-Label distributions

The following tables contain the distributions of the classification labels with respect to each prediction task considered in the manuscript. Each table describes the class distributions within the class stratified train and hold out test splits used for the evaluation of the methods in the manuscript. With the exception of class label 3 in tumour grade for TCGA-HNCS, we are fortunate that the tasks do not show any extreme class imbalances.

Table 1. Distribution of class labels for METABRIC DR Task.

| Class Label | 0 | 1 |
|---|---|---|
| Train + Validation | 1102 | 482 |
| Test | 276 | 120 |

Table 2. Distribution of class labels for METABRIC PAM50.

| Class Label | 0 | 1 | 2 | 3 | 4 |
|---|---|---|---|---|---|
| Train + Validation | 160 | 574 | 390 | 263 | 192 |
| Test | 39 | 144 | 98 | 66 | 48 |

Table 3. Distribution of class labels for METABRIC IC10.

| Class Label | 0 | 1 | 2 | 3 | 4 | 5 | 6 | 7 | 8 | 9 | 10 |
|---|---|---|---|---|---|---|---|---|---|---|---|
| Train + Validation | 208 | 111 | 58 | 232 | 67 | 152 | 68 | 152 | 239 | 116 | 181 |
| Test | 52 | 28 | 14 | 58 | 16 | 38 | 17 | 38 | 60 | 30 | 45 |

Table 4. Distribution of class labels for TCGA-HNCS tumour grade.

| Class Label | 0 | 1 | 2 | 3 |
|---|---|---|---|---|
| Train + Validation | 49 | 243 | 100 | 6 |
| Test | 13 | 61 | 25 | 1 |

Table 5. Class label distributions for TCGA-HNCS 2 Year RFS.

| Class Label | 0 | 1 |
|---|---|---|
| Train + Validation | 163 | 253 |
| Test | 40 | 64 |

## 2 Appendix B: An initial post-hoc functional enrichment study with GINCCo models

We show an example procedure of using GINCCo to identify functionally relevant complex candidates. This showcases a benefit of the GINCCo approach to deterministic and explicit factorisation of the parametric activity functions of named protein complexes (and potentially other higher level modules such as pathways).

For this example we leveraged Integrated Gradients (Sundararajan *et al.*, 2017), a gradient-based attribution method, to estimate the importance of intermediate protein complex nodes in the computation of the target values. We then ranked the protein complexes according to their importance to predictions (i.e. absolute value of the attribution scores) and performed functional enrichment analysis using Enrichr (DisGeNET) to identify enriched pathways of the top protein complexes. For classification of PAM50 on the METABRIC dataset with the GINCCo + DPCLUS combination, we found that the top enriched pathways for the most important protein complex were 1) malignant neoplasm of the breast (q-value: 2.4e-21) and 2) breast carcinoma (q-value: 8.35e-21) as shown in Figure 1. These results suggest that the protein complexes identified by DPCLUS are biologically meaningful and further support our choice for incorporating them as structural inductive biases in our model. More generally, this

© The Author 2020. Published by Oxford University Press. All rights reserved. For permissions, please e-mail: journals.permissions@oup.com 

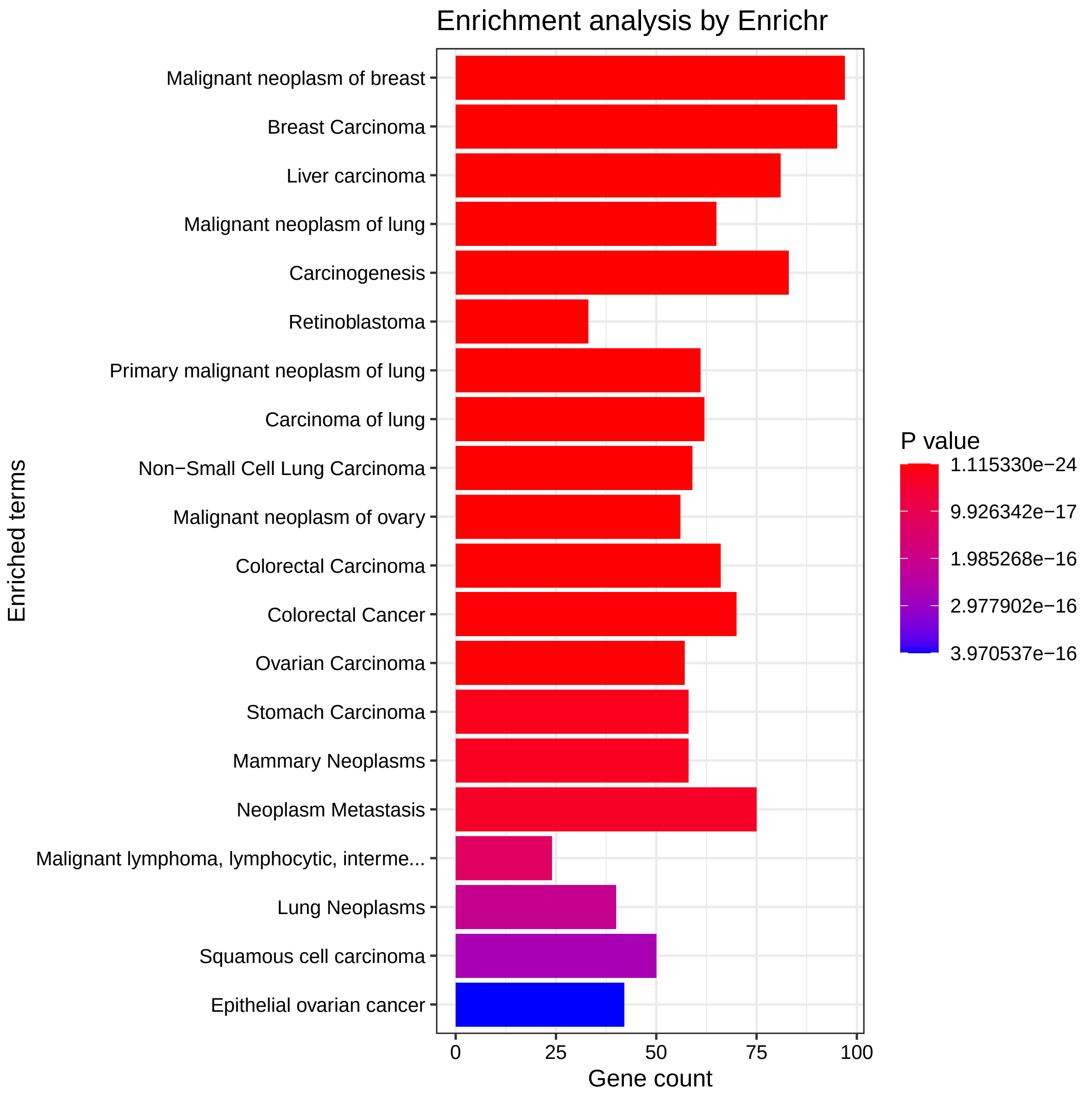


**Fig. 1.** Output results from Enrichr. The ranked list shows how the most influential protein complex in deciding PAM50 classification coming from the GINCCo+DPCLUS model is also highly enriched in carcinoma of the breast, our target disease of interest as well as other carcinomas.

result shows the potential of GINCCo to identify functionally relevant gene-sets given specific phenotype targets and study them through gene set enrichment analysis. A python notebook for this example is made available in our supplementary code.

## 3 Appendix C: Additional metrics

This appendix section contains additional tables of recorded unbalanced accuracy, weighted recall, weighted precision, and weighted F-scores for prediction tasks in the main manuscript.

Table 6. Average hold out unbalanced percentage accuracies for each method over the five class stratified folds of the datasets and tasks.

| | METABRIC | | | TCGA-HNCS | |
|---|---|---|---|---|---|
| Method | DR | PAM50 | IC10 | Tumour Grade | 2 Year RFS |
| MajorityClass | 69.616 + 0.124 | 36.372 + 0.106 | 15.101 + 0.101 | 611.044 + 0.398 | 60.961 + 0.471 |
| SVM | 68.030 + 3.075 | 75.379 + 1.750 | 64.899 + 4.130 | 55.008 + 3.104 | 58.462 + 4.098 |
| FC MLP | 66.262 + 3.727 | 77.760 + 0.897 | 71.868 + 1.223 | 56.830 + 4.098 | 60.769 + 5.245 |
| GraphReg | 68.484 + 0.865 | 37.530 + 2.029 | 13.585 + 1.817 | 58.009 + 4.941 | 58.077 + 2.954 |
| GINCCo + MCODE | 65.253 + 1.972 | 76.138 + 1.808 | 63.788 + 2.637 | 54.618 + 2.647 | 58.076 + 3.016 |
| GINCCo + COACH | 65.606 + 3.107 | 77.861 + 1.506 | 70.152 + 1.816 | 58.234 + 1.455 | 59.231 + 4.411 |
| GINCCo + IPCA | 65.455 + 3.196 | 77.607 + 2.775 | 69.697 + 3.005 | 55.220 + 1.629 | 58.077 + 4.326 |
| GINCCo + DPCLUS | 67.222 + 2.986 | 79.735 + 2.209 | 76.212 + 2.344 | 59.834 + 2.539 | 60.576 + 4.300 |
| RC MLP - R | 66.337 + 2.921 | 74.198 + 9.178 | 63.158 + 11.168 | 54.769 + 6.270 | 58.461 + 3.065 |
| RC MLP - M | 66.925 + 2.603 | 68.341 + 10.365 | 59.778 + 7.610 | 60.544 + 1.843 | 56.607 + 4.533 |

Table 7. Average hold out weighted precision scores for each method over the five class stratified folds of the datasets and tasks.

| | METABRIC | | | TCGA-HNCS | |
|---|---|---|---|---|---|
| Method | DR | PAM50 | IC10 | Tumour Grade | 2 Year RFS |
| MajorityClass | 0.484 + 0.002 | 0.132 + 0.008 | 0.023 + 0.000 | 0.373 + 0.006 | 0.372 + 0.006 |
| SVM | 0.679 + 0.027 | 0.764 + 0.014 | 0.676 + 0.032 | 0.573 + 0.035 | 0.568 + 0.047 |
| FC MLP | 0.651 + 0.030 | 0.786 + 0.012 | 0.721 + 0.009 | 0.563 + 0.041 | 0.606 + 0.043 |
| GraphReg | 0.548 + 0.045 | 0.245 + 0.100 | 0.019 + 0.004 | 0.432 + 0.058 | 0.577 + 0.025 |
| GINCCo + MCODE | 0.639 + 0.010 | 0.769 + 0.018 | 0.619 + 0.057 | 0.538 + 0.028 | 0.577 + 0.029 |
| GINCCo + COACH | 0.643 + 0.012 | 0.782 + 0.012 | 0.681 + 0.028 | 0.561 + 0.057 | 0.591 + 0.036 |
| GINCCo + IPCA | 0.645 + 0.015 | 0.779 + 0.025 | 0.669 + 0.052 | 0.537 + 0.016 | 0.579 + 0.034 |
| GINCCo + DPCLUS | 0.655 + 0.018 | 0.803 + 0.023 | 0.765 + 0.026 | 0.563 + 0.032 | 0.597 + 0.043 |
| RC MLP - R | 0.652 + 0.007 | 0.733 + 0.135 | 0.616 + 0.142 | 0.559 + 0.031 | 0.569 + 0.067 |
| RC MLP - M | 0.628 + 0.039 | 0.627 + 0.156 | 0.496 + 0.114 | 0.552 + 0.030 | 0.551 + 0.095 |

Table 8. Average hold out weighted recall scores for each method over the five class stratified folds of the datasets and tasks.

| | METABRIC | | | TCGA-HNCS | |
|---|---|---|---|---|---|
| Method | DR | PAM50 | IC10 | Tumour Grade | 2 Year RFS |
| MajorityClass | 0.696 + 0.001 | 0.364 + 0.001 | 0.151 + 0.001 | 0.610 + 0.003 | 0.610 + 0.005 |
| SVM | 0.649 + 0.034 | 0.738 + 0.023 | 0.589 + 0.047 | 0.535 + 0.032 | 0.582 + 0.040 |
| FC MLP | 0.634 + 0.028 | 0.764 + 0.015 | 0.704 + 0.013 | 0.563 + 0.039 | 0.602 + 0.051 |
| GraphReg | 0.617 + 0.046 | 0.269 + 0.052 | 0.136 + 0.018 | 0.523 + 0.047 | 0.576 + 0.029 |
| GINCCo + MCODE | 0.639 + 0.010 | 0.752 + 0.018 | 0.629 + 0.028 | 0.538 + 0.020 | 0.579 + 0.029 |
| GINCCo + COACH | 0.639 + 0.023 | 0.772 + 0.019 | 0.692 + 0.021 | 0.572 + 0.011 | 0.589 + 0.044 |
| GINCCo + IPCA | 0.640 + 0.026 | 0.769 + 0.033 | 0.686 + 0.035 | 0.546 + 0.017 | 0.576 + 0.044 |
| GINCCo + DPCLUS | 0.646 + 0.026 | 0.786 + 0.028 | 0.748 + 0.024 | 0.585 + 0.016 | 0.599 + 0.041 |
| RC MLP - R | 0.635 + 0.035 | 0.728 + 0.098 | 0.614 + 0.114 | 0.532 + 0.079 | 0.571 + 0.033 |
| RC MLP - M | 0.633 + 0.051 | 0.656 + 0.113 | 0.575 + 0.079 | 0.586 + 0.019 | 0.558 + 0.060 |

Table 9. Average hold out weighted f-scores scores for each method over the five class stratified folds of the datasets and tasks.

| | METABRIC | | | TCGA-HNCS | |
|---|---|---|---|---|---|
| Method | DR | PAM50 | IC10 | Tumour Grade | 2 Year RFS |
| MajorityClass | 0.571 + 0.001 | 0.194 + 0.001 | 0.039 + 0.000 | 0.463 + 0.004 | 0.462 + 0.006 |
| SVM | 0.663 + 0.012 | 0.751 + 0.018 | 0.629 + 0.039 | 0.553 + 0.031 | 0.584 + 0.043 |
| FC MLP | 0.642 + 0.021 | 0.775 + 0.012 | 0.712 + 0.010 | 0.563 + 0.039 | 0.604 + 0.047 |
| GraphReg | 0.577 + 0.010 | 0.239 + 0.045 | 0.033 + 0.008 | 0.468 + 0.023 | 0.577 + 0.027 |
| GINCCo + MCODE | 0.639 +0.005 | 0.760 + 0.018 | 0.623 + 0.042 | 0.538 + 0.023 | 0.578 + 0.028 |
| GINCCo + COACH | 0.641 + 0.013 | 0.777 + 0.016 | 0.686 + 0.022 | 0.567 + 0.017 | 0.589 + 0.036 |
| GINCCo + IPCA | 0.642 + 0.017 | 0.774 + 0.029 | 0.677 + 0.043 | 0.542 + 0.016 | 0.577 + 0.039 |
| GINCCo + DPCLUS | 0.650 + 0.018 | 0.795 + 0.025 | 0.765 + 0.024 | 0.579 + 0.024 | 0.598 + 0.042 |
| RC MLP - R | 0.643 + 0.021 | 0.729 + 0.120 | 0.614 + 0.131 | 0.541 + 0.064 | 0.569 + 0.054 |
| RC MLP - M | 0.628 + 0.039 | 0.639 + 0.135 | 0.531 + 0.099 | 0.568 + 0.022 | 0.552 + 0.080 |

## 4 Appendix D: Statistical significance tests

We performed (corrected) Student's t-test, using the performance obtained from each train/hold-out split, adjusting the variance as documented in Benavoli *et al.* (2017); Bouckaert and Frank (2004); Nadeau and Bengio (2003). The 2-sided p-values for each target are reported in Table 10. The results show that GINCCo+DPLUCS, while having better a performance in general, the difference is only statistically significant when compared against the MajorityClassifier and GraphReg on the METABRIC tasks.

In contrast there is not a statistically significant difference between the GINCCo variants, the RCMLP and FCMLP, except in the case of predicting IC10. It is really important to note that we have 0.05% of the parameters compared to the FCMLP, and still get comparable (but mostly better) performance. Furthermore ability to perform the GSEA easily where this isn't possible with the other methods we compare against.

Table 10. 2-sided p-values obtained from Student's t-test for each target variable, comparing GINCCO+DPCLUS to the other benchmark methods.

| | METABRIC | | | TCGA-HNCS | |
|---|---|---|---|---|---|
| GINCCO+DPCLUS vs. | DR | PAM50 | IC10 | Tumour Grade | 2 Year RFS |
| MajorityClass | 0.001553 | 6.50E-05 | 1.69E-05 | 0.140736751 | 0.221236 |
| GraphReg | 0.00553 | 3.99E-05 | 1.69E-05 | 0.274251977 | 0.891921 |
| FCMLP | 0.922046 | 0.646701 | 0.265797 | 0.473822691 | 0.124872 |
| RandomMLP_Matched | 0.233221 | 0.002969 | 0.000418 | 0.611882916 | 0.853904 |
| RandomMLP | 0.741983 | 0.096235 | 0.000491 | 0.813377857 | 0.663998 |
| GINCCO+mcode | 0.48463 | 0.377899 | 0.003893 | 0.482437784 | 0.368415 |
| GINCCO+ipca | 0.750309 | 0.629871 | 0.035312 | 0.430973937 | 0.927807 |
| GINCCO+coach | 0.583788 | 0.885773 | 0.027755 | 0.828306483 | 0.345282 |

## References

Benavoli, A., Corani, G., Demšar, J., and Zaffalon, M. (2017). Time for a change: a tutorial for comparing multiple classifiers through bayesian analysis. *Journal of Machine Learning Research*, **18**(77), 1–36.

Bouckaert, R. R. and Frank, E. (2004). Evaluating the replicability of significance tests for comparing learning algorithms. In H. Dai, R. Srikant, and C. Zhang, editors, *Advances in Knowledge Discovery and Data Mining*, pages 3–12, Berlin, Heidelberg. Springer Berlin Heidelberg.

Nadeau, C. and Bengio, Y. (2003). Inference for the generalization error. *Mach. Learn.*, **52**(3), 239–281.

Sundararajan, M., Taly, A., and Yan, Q. (2017). Axiomatic attribution for deep networks. *CoRR*, **abs/1703.01365**.
